# Mortality and hyperkalaemia-associated hospitalisation in patients with chronic kidney disease: comparison of sodium zirconium cyclosilicate and sodium/calcium polystyrene sulfonate

**DOI:** 10.1093/ckj/sfae021

**Published:** 2024-02-20

**Authors:** Chikao Onogi, Yu Watanabe, Akihito Tanaka, Kazuhiro Furuhashi, Shoichi Maruyama

**Affiliations:** Department of Nephrology, Nagoya University, Graduate School of Medicine, Nagoya, Japan; Department of Cell Physiology, Nagoya University, Graduate School of Medicine, Nagoya, Japan; Department of Nephrology, Nagoya University, Graduate School of Medicine, Nagoya, Japan; Department of Nephrology, Nagoya University Hospital, Nagoya, Japan; Department of Nephrology, Nagoya University Hospital, Nagoya, Japan; Department of Nephrology, Nagoya University, Graduate School of Medicine, Nagoya, Japan

**Keywords:** chronic kidney disease, clinical claims database, haemodialysis, hyperkalaemia, potassium adsorbent

## Abstract

**Background:**

Sodium zirconium cyclosilicate (SZC), a novel drug used for treating hyperkalaemia, is effective in reducing serum potassium levels. The effects of potassium adsorbents on the mortality and hyperkalaemia-associated hospitalisation rates remain unclear. We aimed to examine how mortality and hyperkalaemia-associated hospitalisation rates vary with usage of various potassium adsorbents.

**Methods:**

This retrospective study used patients’ data between April 2008 and August 2021 obtained from a large-scale Japanese medical claims database. Consecutive patients with chronic kidney disease (CKD) prescribed potassium adsorbents were enrolled and divided into three groups according to the adsorbent type [SZC, calcium polystyrene sulfonate (CPS), and sodium polystyrene sulfonate (SPS)] and were observed for 1 year. The primary outcome was a composite of mortality and hyperkalaemia-associated hospitalisation.

**Results:**

In total, 234, 54 183, and 18 692 patients were prescribed SZC, CPS, and SPS, respectively. The SZC group showed a higher event-free survival rate than the other two groups. The hazard ratio for the primary outcome in the CPS and SPS groups was similar in the analyses of the subgroups of patients who did not receive renal replacement therapy and those who received haemodialysis. The SZC group had a higher renin-angiotensin-aldosterone system inhibitors (RAASi) continuation rate compared to CPS and SPS groups, the difference being especially significant for SPS.

**Conclusions:**

This real-world study demonstrated the therapeutic effect of SZC in reducing mortality and hyperkalaemia-associated hospitalisations. The high RAASi continuation rate in the SZC group might be a contributing factor for improvement of the primary outcome.

## Graphical Abstract

**Graphical Abstract:**
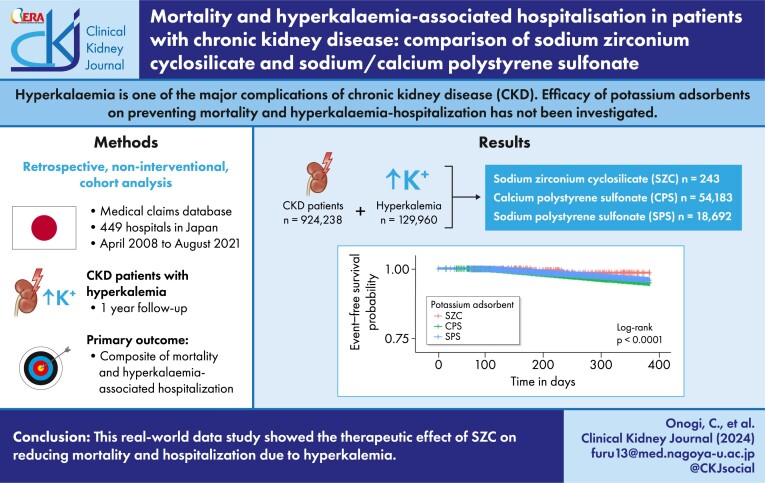

KEY LEARNING POINTS **What was known**:

- Hyperkalaemia is one of the major complications of chronic kidney disease, and it causes life-threatening events such as arrhythmia.
- Potassium adsorbents such as conventional calcium/sodium polystyrene sulfonates and a new compound, sodium zirconium cyclosilicate (SZC), showed clinical efficacy in reducing serum potassium levels in patients with chronic kidney disease with hyperkalaemia.
- Only few studies have examined the role of potassium adsorbents in reducing mortality and hyperkalaemia-associated hospitalization rates in patients with chronic kidney disease.

**This study adds**:

- Patients receiving SZC showed lower mortality and hyperkalaemia-associated hospitalization rates compared to patients receiving calcium/sodium polystyrene sulfonate in this analysis using a Japanese medical claims database.
- Patients receiving SZC showed lower discontinuation rates of renin-angiotensin-aldosterone system inhibitors compared to patients receiving calcium/sodium polystyrene sulfonate.
- Although patients receiving SZC had lower renal functions, they showed similar serum potassium level reductions compared to patients receiving calcium/sodium polystyrene sulfonate.

**Potential impact**:

- SZC may be more effective than calcium/sodium polystyrene sulfonate in reducing mortality and hyperkalaemia-associated hospitalization rates in patients with chronic kidney disease.
- Patients receiving SZC are more likely to continue renin-angiotensin-aldosterone system inhibitors compared to patients receiving calcium/sodium polystyrene sulfonate, which may contribute to better management of chronic kidney disease and heart failure.

## INTRODUCTION

Hyperkalaemia is a major complication of chronic kidney disease (CKD), wherein impaired renal function leads to potassium excretion disorders [1, 2]. Cardiovascular conditions like arrhythmia triggered by hyperkalaemia can lead to life-threatening events [3], and the discontinuation of renin-angiotensin-aldosterone system inhibitors (RAASi) contributes to increased mortality in patients with CKD [4]. Furthermore, high serum potassium levels restrict the intake of fresh fruits and vegetables enriched with alkalis, minerals, and vitamins. Therefore, management of serum potassium levels plays an important role in the treatment of CKD.

Hyperkalaemia is a risk factor for mortality and cardiovascular disease in patients with CKD. Several retrospective studies have reported higher mortality rates, incidence of cardiovascular disease, and hazard ratios in patients with serum potassium levels >5.5 mEq/L [5–8]. Thus, it cannot be stated explicitly that hyperkalaemia has a negative prognostic effect on mortality and cardiovascular events in patients with CKD, though studies have highlighted a requirement for maintaining serum potassium levels in the normal range [9].

Management of hyperkalaemia is conventionally initiated with dietary restriction of potassium intake [10], followed by prescription adjustments to prevent drug-induced hyperkalaemia, and correction of metabolic acidosis. Moreover, potassium adsorbents also play an important therapeutic role. Positive ion exchange resins are widely used as potassium adsorbents; calcium polystyrene sulfonate (CPS) and sodium polystyrene sulfonate (SPS) have been reported to lower serum potassium levels [11, 12]. However, few studies have discussed the effect of the above-mentioned compounds in lowering mortality and re-hospitalisation rates. Sodium zirconium cyclosilicate (SZC), a non-polymeric agent, has recently emerged as a new potassium adsorbent [13–16].

This study aimed to examine how mortality and hospitalisation rates associated with hyperkalaemia recurrence vary with the prescription of various potassium adsorbents in a Japanese medical claims database. To the best of our knowledge, this is the first report to examine the therapeutic effect of potassium adsorbents on the mortality and hospitalisation rates in patients with CKD and hyperkalaemia using real-world data.

## MATERIALS AND METHODS

### Study design

This was a non-interventional, retrospective cohort study using the Japanese medical claims database obtained from Medical Data Vision Co., Ltd (MDV). Data were collected from 36 690 000 patients in 449 hospitals between April 2008 and August 2021. It included individual records of procedures, prescriptions, surgeries, hospitalisations, and laboratory data.

Approval for database assessment was obtained from the Ethics Committee of the International Review Board of Nagoya University Hospital (approval number: 2021–0350). The requirement for informed consent was waived due to the anonymised nature of the claims database.

### Study population

**Figure 1:**
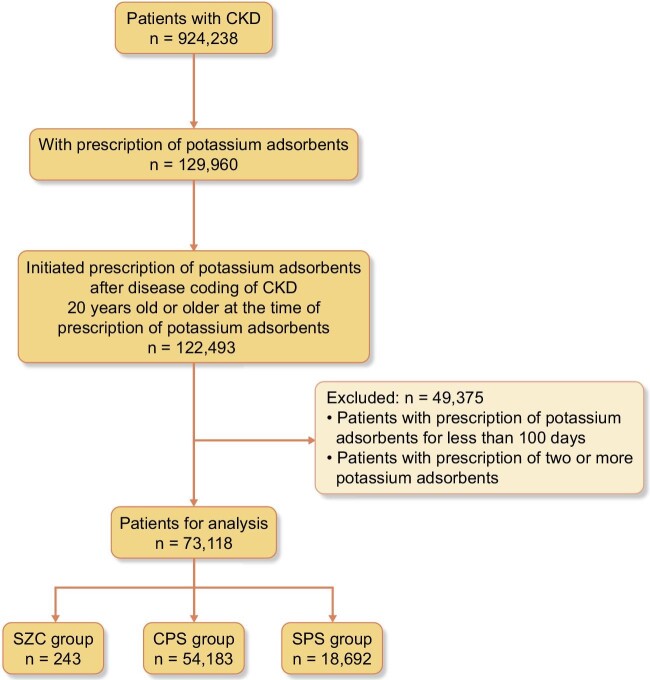
Flowchart depicting inclusion and exclusion criteria. CKD, chronic kidney disease; CPS, calcium polystyrene sulfonate; SPS, sodium polystyrene sulfonate; SZC, sodium zirconium cyclosilicatex.

From the claims database, we extracted data of patients with CKD codes (924 238 patients); among these, patients aged ≥20 years were selected (Table S1, see online supplementarymaterial). To collect data on patients with CKD with hyperkalaemia, we utilised the prescription history of potassium adsorbents, and only those prescribed potassium adsorbents were the targets of our analysis. Patients who started treatment with potassium adsorbents (SZC, CPS, or SPS) during the observation period and received consecutive prescriptions for at least 100 days were enrolled. The observation period was 1 year. Only the first prescription during the observation period was included in the analysis; subsequent prescriptions as well as prescriptions from prior to the observation period were not regarded as new users and were not analysed. Patients treated with more than one potassium adsorbent were also excluded. Finally, the patients were divided into three groups according to the prescribed potassium adsorbents (SZC, CPS, or SPS). Fig. 1 shows a flowchart of patient registration.

### Assessment of outcome

The primary endpoint was a composite of mortality and hospitalisation with a diagnosis of hyperkalaemia. Death was assessed using discharge summaries. Hospitalisation caused by hyperkalaemia was assessed from the registration of the disease code for hyperkalaemia as a primary disease at the time of admission.

Secondary endpoints were as follows: (A) prescription rate of RAASi [angiotensin II receptor blocker (ARB) and angiotensin-converting enzyme inhibitor (ACEi)] in the group of patients using RAASi at the start of potassium adsorbent prescription; (B) prescription rate of laxatives; and (C) changes in serum potassium levels for patients in whom laboratory data were stored in the claims database.

### Statistical analyses

Numerical data were described using the mean and standard deviation. Categorical data are presented as percentages. Baseline characteristics were compared between each treatment group using Pearson's chi-squared test, Kruskal–Wallis rank sum test, or Fisher's exact test, as appropriate. For the primary outcome, Kaplan–Meier curves were generated and log-rank tests were performed to compare the total survival rates of each treatment group. Multivariate analyses applying the Cox hazard model were performed using hazard ratios and are graphically presented as forest plots. Hazard ratios were adjusted for several clinical factors such as age, sex, history of hypertension, diabetes, heart failure, ischaemic heart disease, cerebrovascular disease, and prescription of ARB, ACEi, beta-blockers, sodium-glucose cotransporter-2 inhibitors (SGLT2), and mineralocorticoid receptor antagonists (MRA) at the start of the observation period. These analyses were also performed in subgroups of patients without renal replacement therapy (RRT) and with maintenance haemodialysis (MHD). To assess the additional outcomes of (A) and (B), the patients who were prescribed at the beginning of the observation period were extracted and the prescribing status at the end of the observation period was compared by Fisher's exact test for each treatment group. To avoid multiple comparison problems, we adopted the Bonferroni correction. Changes in serum potassium levels are presented as boxplots along a timeline.

## RESULTS

### Baseline characteristics of patients

From the medical claims database, we identified 243, 54 183, and 18 692 patients who were initiated on SZC, CPS, and SPS, respectively (Fig. 1). The baseline characteristics are presented in Table 1. In each group, 65–66% were men with an age range of 72–74 years, with significant differences (*P* < 0.001). Compared to patients initiated with CPS and SPS, those receiving SZC had a higher percentage of MHD patients (46% vs. 8.2% and 13% for CPS and SPS, respectively). Furthermore, a history of hypertension was common in all the groups. Approximately half of the patients in each group had a history of heart failure (53, 49, and 44% in SZC, CPS, and SPS, respectively). In baseline prescription, approximately half of patients were prescribed ARB (47, 49, and 51% for SZC, CPS, and SPS, respectively), which is a major cause of drug-induced hyperkalaemia. ACEi, another RAASi, was prescribed to some patients (3.3, 9.6, and 8.3% for SZC, CPS, and SPS, respectively), although the prescription rate was lower than that for ARB. As for other agents, across all groups, beta blocker prescription rates were limited up to 30% and <10% for MRA. In addition, the percentage of patients who did not complete the 1-year observation period was notably higher in the SZC group. The baseline characteristics in the subgroup of patients without RRT showed trends similar to those of the whole group. In contrast, no significant differences were observed when medical histories and baseline prescriptions were compared in the subgroup analysis of patients receiving MHD.

**Table 1:** Baseline characteristics

Characteristics	SZC, *n* = 243	CPS, *n* = 54 183	SPS, *n* = 18 692	*P*-value
Men, *n* (%)	160 (66)	35 059 (65)	12 411 (66)	<0.001
Age, y, mean (SD)	72 (12)	74 (12)	72 (12)	<0.001
RRT condition, *n* (%)				
no RRT	130 (53)	49 466 (91)	16 150 (86)	
MHD	111 (46)	4455 (8.2)	2403 (13)	
PD	2 (0.8)	262 (0.5)	139 (0.7)	
Disease history, *n* (%)				
HT	228 (94)	49 104 (91)	17 183 (92)	<0.001
DM	161 (66)	34 471 (64)	11 609 (62)	<0.001
Af and AFL	32 (13)	8668 (16)	2137 (11)	<0.001
HF	129 (53)	26 730 (49)	8224 (44)	<0.001
IHD	32 (13)	8453 (16)	2328 (12)	<0.001
CD	73 (30)	14 301 (26)	4583 (25)	<0.001
HPL	144 (59)	31 098 (57)	10 731 (57)	0.840
Medication history at the start of observation, *n* (%)				
ACEi	8 (3.3)	5215 (9.6)	1555 (8.3)	<0.001
ARB	113 (47)	26 281 (49)	9491 (51)	<0.001
Beta blocker	74 (30)	15 702 (29)	4407 (24)	<0.001
SGLT2i	16 (6.6)	1334 (2.5)	312 (1.7)	<0.001
MRA	12 (4.9)	4048 (7.5)	972 (5.2)	<0.001
Laxatives	44 (18)	10 502 (19)	3246 (17)	<0.001
Medication history at the end of observation, *n* (%)				
ACEi	7 (2.9)	4163 (7.7)	1218 (6.5)	<0.001
ARB	108 (44)	21 713 (40)	7891 (42)	<0.001
Beta blocker	74 (30)	14 545 (27)	4128 (22)	<0.001
SGLT2i	21 (8.6)	1618 (3.0)	346 (1.9)	<0.001
MRA	12 (4.9)	2838 (5.2)	730 (3.9)	<0.001
Laxatives	38 (16)	9649 (18)	2833 (15)	<0.001
Primary outcome, *n* (%)				
Composite	2 (0.8)	2489 (4.6)	703 (3.8)	<0.001
Death	2 (0.8)	2233 (4.1)	617 (3.3)	
Hyperkalaemia-associated hospitalisation	0 (0)	256 (0.5)	86 (0.5)	

ACEi: angiotensin converting enzyme inhibitor; Af: atrial fibrillation; AFL: atrial flutter; ARB: angiotensin II receptor blocker; CD: cerebrovascular disease; CPS: calcium polystyrene sulfonate; DM: diabetes mellitus; HD: maintenance haemodialysis; HF: heart failure; HPL: hypercholesterolaemia; HT: hypertension; IHD: ischaemic heart disease; MRA: mineralocorticoid receptor antagonist; PD: peritoneal dialysis; RRT: renal replacement therapy; SGLT2i: sodium-glucose cotransporter 2 inhibitor; SPS: sodium polystyrene sulfonate; SZC: sodium zirconium cyclosilicate.

### Primary outcome: mortality and hospitalization with hyperkalaemia diagnosis

**Figure 2:**
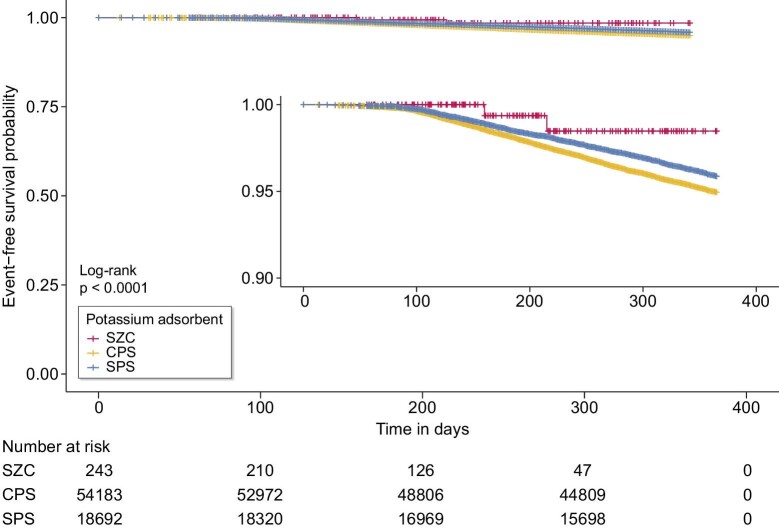
Kaplan–Meier curve of event-free survival rates of composite of mortality and hyperkalaemia-associated hospitalization rate. The inset shows the same data on an expanded y-axis. CPS, calcium polystyrene sulfonate; SPS, sodium polystyrene sulfonate; SZC, sodium zirconium cyclosilicate.

**Figure 3:**
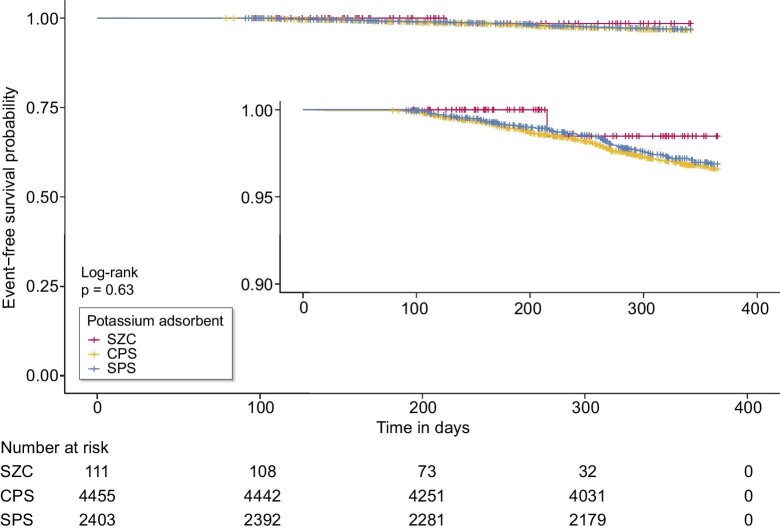
Kaplan–Meier curve of event-free survival rates of composite of mortality and hyperkalaemia-associated hospitalization rate limited to a subgroup of patients with maintenance haemodialysis. The inset shows the same data on an expanded y-axis. CPS, calcium polystyrene sulfonate; SPS, sodium polystyrene sulfonate; SZC, sodium zirconium cyclosilicate.

The ratio of events, defined as the primary outcome, was lower in SZC group than in other groups (0.8% for SZC compared with 4.6 and 3.8% for CPS and SPS, respectively; Table 1). Fig. 2 shows the Kaplan–Meier curves of event-free survival rates for mortality and hyperkalaemia-associated hospitalisation. Among all groups, the overall survival rates were >80% throughout the observation period with a significantly higher survival rate in the SZC group (*P* < 0.001, log-rank test). Subgroup analysis limited to MHD patients showed no significant difference in survival rates, but the SZC group tended to have a higher survival rate compared to the other groups (Fig. 3).

**Figure 4:**
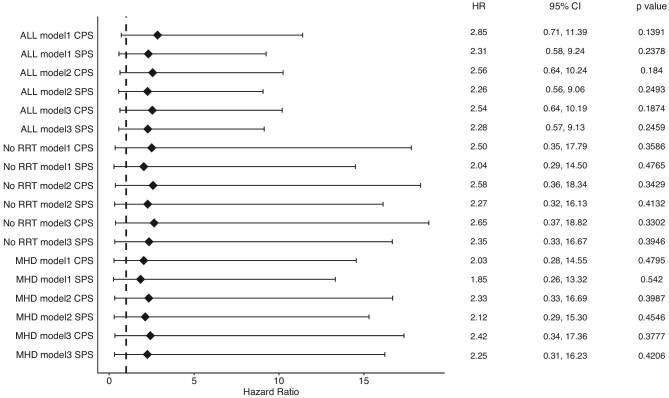
Forest plot shows hazard ratio of primary outcome. Reference group is sodium zirconium cyclosilicate group. Notes: ALL, analysis for all patients; MHD, analysis for a subgroup of patients with maintenance haemodialysis; No RRT, analysis for a subgroup of patients without renal replacement therapy; Model 1, not adjusted; Model 2, adjusted by age and sex; Model 3, adjusted by age, sex, history of hypertension, diabetes, heart failure, ischemic heart disease, cerebrovascular disease, prescription of angiotensin II receptor blocker, angiotensin convertor enzyme inhibitor, beta blocker, sodium-glucose cotransporter-2 inhibitor, and mineralocorticoid receptor antagonist. CPS, calcium polystyrene sulfonate; SPS: sodium polystyrene sulfonate.

Hazard ratios (HR) were calculated for the CPS and SPS groups for comparison with the SZC group (Fig. 4) by using the three models adjusted for various clinical factors: Model 1 includes no adjustment, so the model is equal to univariate analysis; Model 2 includes age and sex as adjustment factors; Model 3 uses history of hypertension, diabetes, heart failure, ischaemic heart disease, cerebrovascular disease, prescription of ARB, ACEi, beta blocker, SGLT2, and MRA at the start of the observation period in addition to Model 2. Although no significant differences were found using either explanatory of the variable model, the CPS and SPS groups showed high HRs (2.28–2.85). Analyses of the subgroups of patients both without RRT and with MHD also showed high HRs in the CPS and SPS groups, but no significant differences were observed (Fig. 4).

### Secondary endpoint (A): Prescription rate of RAASi

RAASi are key drugs used for treating patients with CKD and heart failure. Thus, it is important to continue prescribing RAASi while preventing drug-induced hyperkalaemia. In patients who were continuously prescribed RAASi at the start of the observation period, we assessed the prescription duration and compared the three potassium adsorbent groups (Table 2). Prescription retention rates of RAASi were compared between the groups using Fisher's exact test. The SZC group had a significantly higher RAASi prescription continuation rate compared to the SPS group (*P* = 0.031). Although no significant differences were found for the other combinations (*P* = 0.054 for SZC vs. CPS and *P* = 0.092 for CPS vs. SPS); the SZC group had a lower discontinuation rate than the CPS group.

**Table 2:** Cross table for RAASi continuity

Prescription status of RAASi at the end of the observation period	SZC *n* = 118	CPS *n* = 30 205	SPS *n* = 10 495
Without prescription, *n* (%)	18 (15)	7427 (25)	2692 (26)
With prescription, *n* (%)	100 (85)	22 778 (75)	7803 (74)

RAASi includes angiotensin converting enzyme inhibitor and angiotensin II receptor blocker. Analysed populations were restricted to patients who prescribed RAASi at the beginning of the observation period.

### Secondary endpoint (B): Prescription rate of laxatives

Constipation is known as the adverse effect of positive ion-exchange resins which is composed of polymers, such as CPS and SPS. Aggravated constipation can result in obstruction or perforation of the intestinal tract [17]. One of the advantages of non-polymer adsorbents, such as SZC, is the lower incidence of constipation than with polymer adsorbents. We evaluated the prescription rates of laxatives and compared the three potassium adsorbent groups (Table 3). Fisher's exact test for the prescription retention rate in each pair of adsorbent groups showed no significant differences among all combination pairs selected from the three groups (*P* = 1,1 and 0.58 SZC, SZC vs SPS and CPS vs SPS, respectively).

**Table 3:** Cross table for laxatives continuity

Prescription status of laxatives at the end of the observation period	SZC *n* = 44	CPS *n* = 10 502	SPS *n* = 3246
Without prescription, *n* (%)	19 (43)	4354 (41)	1388 (43)
With prescription, *n* (%)	25 (57)	6148 (59)	1858 (57)

Analysed populations were restricted to patients who prescribed laxatives at the beginning of the observation period.

### Secondary endpoint (C): Changes in serum potassium levels

**Figure 5:**
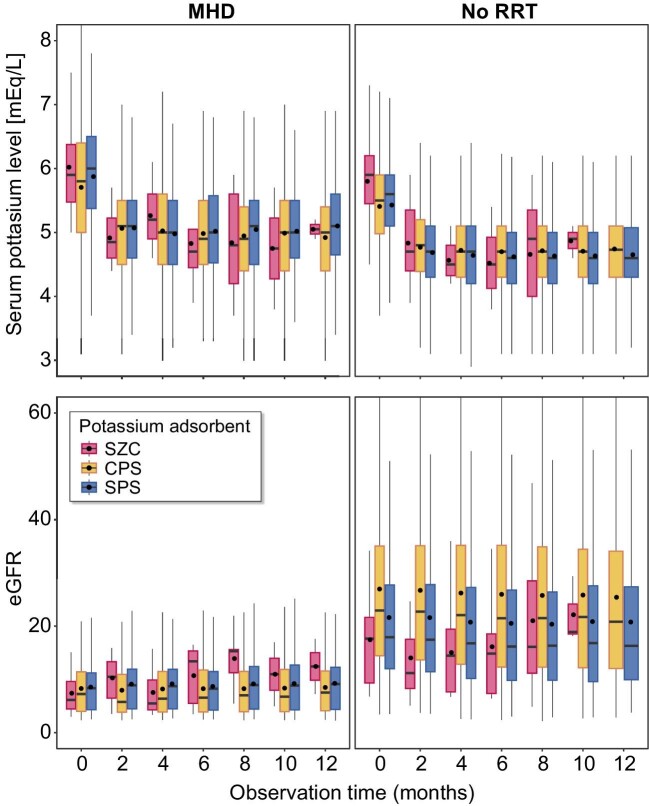
Box plots of time series of serum potassium levels. CPS, calcium polystyrene sulfonate; M, months; MHD, analysis for a subgroup of patients with maintenance haemodialysis; No RRT, analysis for a subgroup of patients without renal replacement therapy; SPS, sodium polystyrene sulfonate; SZC, sodium zirconium cyclosilicate.

To assess the efficacy of each potassium adsorbent, the time series of serum potassium levels were compared for each potassium adsorbent group. Only patients with laboratory data in the claims database were included in the analysis. Fig. 5 shows boxplots of the changes in serum potassium levels and estimated glomerular filtration rate (eGFR) in each group. In all the adsorbent groups, the serum potassium levels of patients in the quartile ranges were within the normal range within approximately 2 months of the start of the prescription. In the subgroup of patients without RRT, although no significant differences were observed, patients in the SZC group tended to have a lower eGFR than those in the other two groups.

## DISCUSSION

In this study, patients with CKD and hyperkalaemia were divided into three groups based on the initiation of potassium adsorbents (SZC, CPS, and SPS). We found a significant decrease in the composite of mortality and hyperkalaemia-associated hospitalisation rates in the SZC group. SZC users showed lower total mortality than the other medication groups. The effect of SZC on reducing mortality is noteworthy because the management of serum potassium levels is aimed at decreasing mortality. There were no hospitalisations due to the recurrence of hyperkalaemia in the SZC group. In the patient population, only a small number of patients were initiated with SZC treatment as it is a new and expensive drug. Therefore, not enough events may have been observed with SZC.

In the multivariate analysis, three models (a univariate model and two adjusted variable models) were applied to calculate the HRs. In all models, although they were not significantly different, the increased HRs in the CPS and SPS for SZC suggest a benefit of SZC on the primary outcome. In addition, the SZC superiority on the primary outcome was indicated even after adjusting for various clinical factors, which showed significant differences in baseline characteristics. Moreover, it is possible that a larger number of patients in the analysis demonstrated the efficacy of SZC with a significant difference.

Regarding the baseline characteristics, the group of patients initiating SZC included more MHD patients than the other groups, and the univariate analysis of MHD patients showed no significant differences among the groups. This suggests that potassium removal by MHD may contribute strongly to the SZC group in addition to the effect of the adsorbent. However, the MHD patients showed higher event rates in the CPS and SPS groups than in the SZC group as indicated by the Kaplan–Meier curve. In addition, the multi-variate analysis limited to MHD patients also displayed an increase in the HRs in the CPS and SPS group. These results suggest that SZC is superior to the other two agents even when the effect of potassium removal by MHD is taken into account.

Although there is strong evidence that inhibition of the renin-angiotensin-aldosterone system has beneficial effects on the prognosis of proteinuria [18], diabetic nephropathy [19, 20], and heart failure [21–24], hyperkalaemia is a major cause of RAASi discontinuation. Prescribing SZC is expected to improve the prescription rate of RAASi in CKD patients. The RAASi discontinuation rate was lower in the SZC group than in the other two groups at the end of the observation period. Fisher's exact test showed significantly higher prescription rates of RAASi in the SZC group than in the SPS group. These results indicated that SZC has a preventive effect against drug-induced hyperkalaemia. As discussed below, the SZC group tended to have poorer renal function than the other two groups. In actual clinical practice, RAASi is discontinued for various reasons, such as low eGFR, hyperkalaemia, hypotension, low carbonate level, and AKI-related hospitalisation [4]. In this study, it was unclear which factors predominantly contributed to the discontinuation of RAASi use. However, it should be noted that the SZC group had a higher RAASi retention rate than the other two groups despite the inclusion of more patients with impaired renal function.

SZC is composed of non-polymer molecules and is expected to be associated with a lower incidence rate of gastrointestinal disorders, particularly constipation. Contrary to expectations, we did not observe any differences in the prescription rates of laxatives between the groups. As noted, one of the reasons for these ambiguous results may be the small number of patients in the SZC group. For the inclusion criteria, we adopted a prescription continuity of 100 days for each potassium adsorbent. Therefore, patients with persistent constipation may not meet the continuity criteria and may have been excluded from our study. In real-world practice, the dose of potassium adsorbents tends to be lower than that used in randomized controlled trials [25]. In clinical practice, the dose of potassium adsorbents is adjusted within a range that achieves a certain level of efficacy while avoiding adverse effects. This makes it difficult to observe differences in the prescription rates of laxatives.

The effect of treatment on serum potassium levels was evaluated in patients whose laboratory data were recorded in a claims database. In all groups, the serum potassium levels were maintained around the control target range within 2 months of initiating the prescription of each absorbent. Although the prescribed dose was not discussed, all adsorbents provided sufficient therapeutic effects to achieve the management target. In the analysis of patients who did not receive RRT, a lower eGFR was observed in the SZC group throughout the observation period. These results indicated that SZC tends to be prescribed to patients with severe CKD. The achievement of the control target range of serum potassium levels implies that SZC has shown sufficient therapeutic effect in patients with severe CKD.

A recent study suggested that discontinuation of RAASi did not lead to an increase of eGFR in patients with advanced and progressive CKD [26]. Although the study above did not discuss hyperkalaemia as an adverse event, the study results may support continued prescription of RAASi while allowing serum potassium levels to rise to near the upper limit of a normal range in clinical practice. Especially in patients with severe CKD, SZC may contribute to both management of serum potassium levels and continuation of RAASi prescriptions, which may be one reason for the improvement of the primary outcome in the SZC group.

Two other possible reasons that contributed to improve the primary outcome in the SZC are listed below. First, although the continuation rate of laxatives showed no significant differences, SZC, a non-polymeric potassium adsorbent, may have reduced the incidence of death caused by intestinal perforation and intestinal obstruction. Second, because our database contains multicentre data, it is possible that hospitals aggressively using novel drugs such as SZC may be excellent at management of CKD. The difference in hospital quality may be manifested as improvement of the primary outcome.

This study had limitations. First, this was a retrospective study, and patients were recruited at the time of planning this study, which did not have prospective data; therefore, the number of patients may be insufficient. Second, the SZC is a novel and expensive potassium adsorbent that is used for a limited number of patients. Therefore, the number of deaths and hospitalisations may not be sufficiently observable among patients in the SZC group. In terms of assessment of discontinuation rates of RAASi, inclusion of large numbers of patients is preferable in this type of study. In future research, more patient data should be analysed for a more detailed discussion. In the analysis of secondary endpoints, we discussed changes in serum potassium levels. However, the analysis was only applicable to a subset of patients because laboratory data were only stored for some patients in the claims database. Third, creatinine data were not available for all patients; therefore, the CKD stage was unclear.

In conclusion, we determined whether mortality and hyperkalaemia-associated hospitalisation rates in patients vary with the type of potassium adsorbent prescribed. Patients in the SZC group had lower mortality and hyperkalaemia-associated hospitalisation rates than those in the CPS and SPS groups.

## Supplementary Material

sfae021_Supplemental_Table

## Data Availability

The data analysed in the study was obtained from Medical Data Vision Co., Ltd, under an agreement not to disclose the data to outside parties.
