# Supplementary material for: Mortality and hyperkalaemia-associated hospitalisation in patients with chronic kidney disease: comparison of sodium zirconium cyclosilicate and sodium/calcium polystyrene sulfonate
Source: Clin Kidney J. 2024 Feb 20;17(2):sfae021. doi: 10.1093/ckj/sfae021 (PMC10894033; doi:10.1093/ckj/sfae021)
Supplement: sfae021_Supplemental_Table [file sfae021_supplemental_table.docx]

**Supplementary Table 1.** ICD10 code of diseases

| Disease name | ICD10 code |
| --- | --- |
| CKD | N181, N182, N183, N184, N185, and N189 |
| HT | I10, I11, I12. I13, I14, and I15 |
| DM | E10, E11, E12, E13, and E14 |
| Af and AFL | I48 |
| HF | I50 and I110 |
| IHD | I21, I22, I23, I24, and I25 |
| CD | I60, I61, I62, I63, I64, I65, I66, I67, I68, I69, and G45 |
| HPL | E780, E781, E782, E783, E784, E785, E786, E787, E788, and E789 |

CKD: Chronic kidney disease; HT: Hypertension; DM: Diabetes mellitus; Af: Atrial fibrillation; AFL: Atrial flutter; HF: Heart failure; IHD: Ischaemic heart disease; CD: Cerebrovascular disease; HPL: Hypercholesterolaemia
